# Meta‐analysis of PD‐(L)1 inhibitor plus chemotherapy versus chemotherapy as first‐line treatment in extensive‐stage small‐cell lung cancer

**DOI:** 10.1002/cam4.6433

**Published:** 2023-08-11

**Authors:** Jiangyue Lu, Xiao Lei, Pei Zhang, Lehui Du, Zhibo Zhang, Baolin Qu

**Affiliations:** ^1^ Department of Radiation Oncology The Fifth Medical Center of Chinese PLA General Hospital Beijing China; ^2^ Medical School of Chinese PLA Beijing China; ^3^ The 78th Group Army Hospital of Chinese PLA Mudanjiang China

**Keywords:** chemotherapy, extensive‐stage, immunotherapy, meta‐analysis, small‐cell lung cancer

## Abstract

**Background:**

Immunotherapy targeting programmed death 1(PD‐1) and its ligand (PD‐L1) has been successful in extensive‐stage small cell lung cancer (ES‐SCLC). However, first‐line PD‐(L)1 inhibitor combined with chemotherapy (immunochemotherapy) versus chemotherapy has not been well studied.

**Methods:**

Randomized controlled trials had been searched from PubMed, Embase, and the Cochrane Library until December 29, 2022. Randomized effect consistency models were applied for estimating the pooled hazard ratios (HRs) and odds ratios (ORs). Study outcomes included overall response rate (ORR), progression‐free survival (PFS), overall survival (OS), 6‐month and 1‐year disease progression rate, 1‐year and 2‐year mortality rate, and Grade ≥3 adverse events (AEs).

**Results:**

Six eligible trials with 2600 ES‐SCLC patients were included. Compared with chemotherapy, immunochemotherapy significantly improved ORR (OR 1.32, 95% CI 1.07–1.63; *p* = 0.01), PFS (HR 0.68, 95% CI 0.58–0.78; *p* < 0.001), and OS (HR 0.72, 96% CI 0.66–0.78, *p* < 0.001) without increasing Grade ≥3 AEs (*p* = 0.07). Compared with patients with chemotherapy, the 6‐month disease progression rate was reduced by 0.39 (*p* = 0.01) and the 1‐year disease progression rate was reduced by 0.75 (*p* < 0.001), the 1‐year mortality rate was reduced by 0.33 (*p* < 0.001) and the 2‐year mortality rate was reduced by 0.50 (*p* < 0.001) respectively in patients with immunochemotherapy. However, patients with brain metastases failed to prolong PFS and OS from immunochemotherapy (*p* > 0.05).

**Conclusion:**

Compared with chemotherapy, PD‐(L)1 inhibitor plus chemotherapy as first‐line treatment could improve the efficacy and prognosis of ES‐SCLC patients without more serious side effects. However, more research is needed to validate these results.

## 1 INTRODUCTION

Small cell lung cancer (SCLC), accounting for approximately 15% of newly diagnosed lung cancers, is a highly progressive and lethal subtype with characteristics of rapid tumor growth, early metastasis, and recurrence.^1^, ^2^, ^3^ Most patients were initially diagnosed with extensive‐stage small cell lung cancer (ES‐SCLC).^4^ For decades, platin‐based chemotherapy has been the standard first‐line treatment for ES‐SCLC.^5^ Despite a good response to chemotherapy, most patients relapse rapidly, and the median survival remained less than 1 year in most clinical trials.^6^, ^7^, ^8^ Therefore, new drugs and treatment strategies are urgently needed to change the current treatment dilemma.

Recently, immunotherapy targeting programmed death (ligand)‐1 [PD‐(L)1] has demonstrated great antitumor activity and has been applied for first‐line treatment of patients with ES‐SCLC. IMpower 133 first reported that first‐line PD‐L1 inhibitor (atezolizumab) plus chemotherapy versus chemotherapy improved the prognosis in patients with ES‐SCLC.^9^ Based on these results, this combination strategy has been recommended as one option in the first‐line treatment of ES‐SCLC. However, the long‐term survival and safety of this combination treatment strategy have not been well investigated, and its clinical benefits in brain metastases, liver metastases, PD‐L1, and lactate dehydrogenase (LDH) subgroups are still unclear.

Therefore, we conducted this meta‐analysis to determine whether first‐line PD‐(L)1 inhibitor plus chemotherapy (immunochemotherapy) was superior to chemotherapy in the first‐line treatment of ES‐SCLC. Moreover, we compared immunochemotherapy with chemotherapy in the subgroup analysis.

## 2 METHODS

### 2.1 Data sources and search strategy

Studies were identified from PubMed, Embase, and the Cochrane Library up to December 29, 2022. The search terms were (“immune checkpoint” OR “programmed cell death‐1” OR “programmed cell death ligand‐1” OR PD‐1 OR nivolumab OR pembrolizumab OR opdivo OR keytruda OR PD‐L1 OR atezolizumab OR durvalumab OR imfinzi OR tecentriq OR avelumab OR adebrelimab OR serplulimab OR cemiplimab OR envafolimab OR pidilizumab) AND (chemotherapy OR cisplatin OR carboplatin OR cis‐platinum OR platinum OR etoposide) AND (carcinoma, small cell lung [MeSH] OR sclc) AND (“extensive‐stage” OR “extensive stage” OR “extensive‐disease” OR “extensive disease” OR “extensive”) AND (“first‐line” OR “untreated” OR “treatment naive” OR “chemo naive” OR “front line”) AND (“randomized controlled trial” OR RCT OR “controlled clinical trial” OR randomized OR randomly OR trial). The details were included in Appendix S1.

### 2.2 Study inclusion and extraction

Studies were included according to (I) ES‐SCLCs were diagnosed by histology or cytology; (II) studies involved comparing first‐line PD‐(L)1 plus chemotherapy with chemotherapy; (III) studies designed as randomized controlled trials (RCTs) with phase II or III; (IV) studies reported treatment outcomes including objective response rate (ORR), progression‐free survival (PFS), overall survival (OS), and Grade ≥3 adverse events (AEs). Studies were excluded if (I) duplicate publications without the latest published data; (II) reviews, protocols, case reports, meta‐analyses, or letters; (III) studies were retrospective or single‐arm; (IV) studies with data unavailable.

### 2.3 Data extraction and quality evaluation

Two researchers (Jiangyue Lu and Zhibo Zhang) independently reviewed articles and extracted data from each eligible study, and disputes were resolved through discussion. The extracted information included first author, year of publication, study title, study design, number of patients, study phase, treatment regiments, study and control group characteristics including age, smoke status, Eastern Cooperative Oncology Group performance status (ECOG PS), brain metastases, liver metastases, PD‐L1, LDH, number of patients with complete response, partial response, and Grade ≥3 AEs, 6‐month and 1‐year disease progression rate, 1‐year and 2‐year mortality rate, and hazard ratios (HRs) and 95% confidence intervals (CIs) for PFS and OS.

Two researchers (Jiangyue Lu and Zhibo Zhang) evaluated the quality of included studies independently using the Cochrane “risk of bias” tool.^10^ Disputes were settled through discussion. Quality assessments were provided in Appendix S2.

### 2.4 Statistical analysis

All analysis results were performed using Review Manager 5.4. Cumulative HRs were calculated using random‐effect models. Odds ratios (ORs) and 95% CIs were applied for ORR, 6‐month disease progression rate, 1‐year disease progression rate, 1‐year mortality rate, 2‐year mortality rate, and Grade ≥3 AEs. HRs with 95% CIs were applied for PFS and OS. Statistical heterogeneity was assessed using *I* ^2^ and *p* values, with *I* ^2^ ≥ 50% or *p* < 0.1 indicating significant heterogeneity among studies. Each test was two‐sided, and a *p* < 0.05 was considered significant.

## 3 RESULTS

### 3.1 Literature selection

**FIGURE 1:**
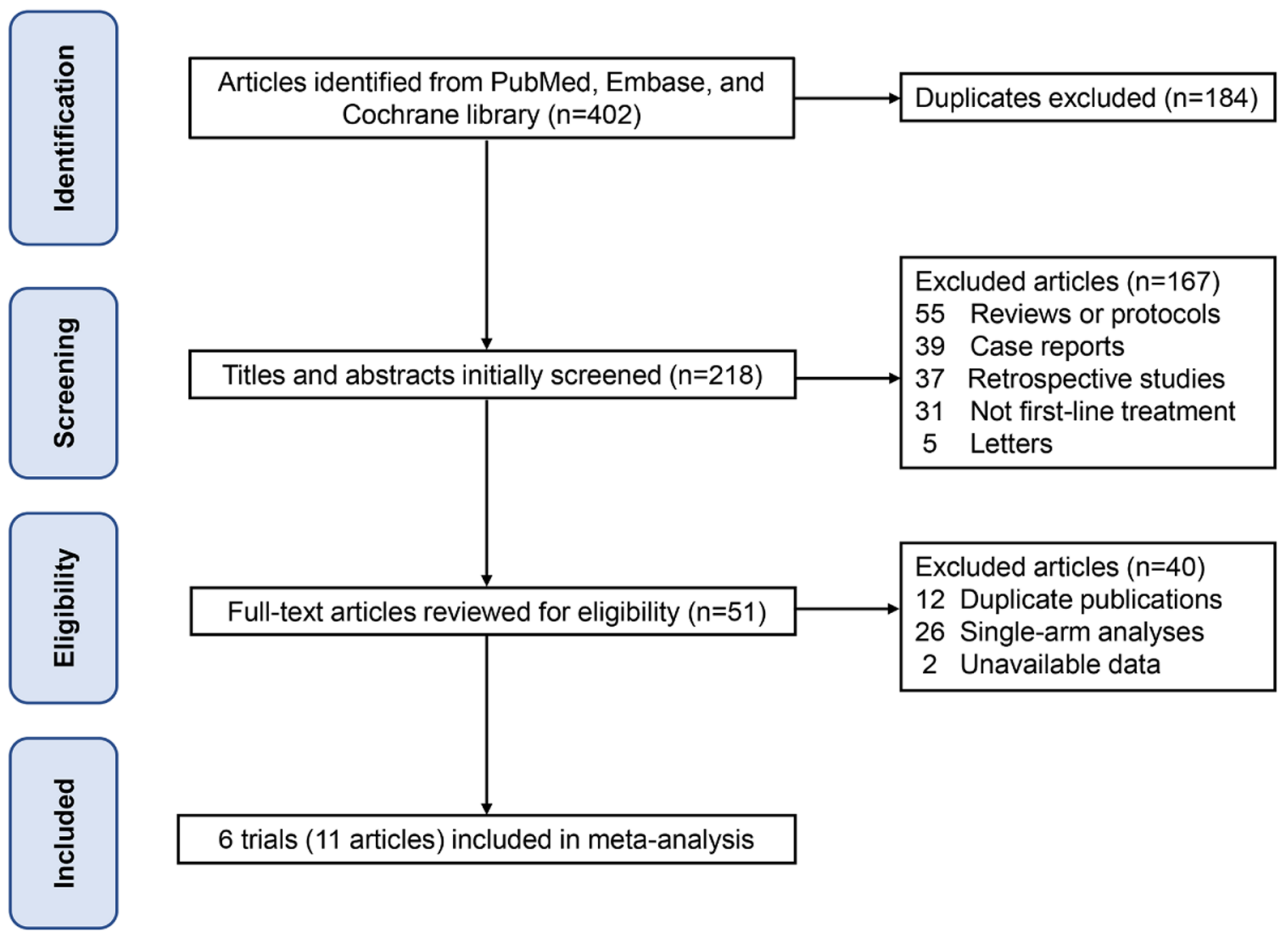
Flowchart of eligible studies selection.

The flowchart of eligible studies selection was shown in Figure 1. Six eligible RCTs (11 articles) were finally included in this study, involving 2600 ES‐SCLC patients who received either immunochemotherapy (1204 patients) or chemotherapy (1396 patients). Seven articles^9^, ^11^, ^12^, ^13^, ^14^, ^15^, ^16^ reported three trials on PD‐L1 inhibitors, and four articles^17^, ^18^, ^19^, ^20^ reported three trials on PD‐1 inhibitors. All six trials reported outcomes of ORR, PFS, OS, and Grade ≥3 AEs. Four trials reported brain and liver metastases subgroup results, three trials reported PD‐L1 subgroup results, and two trials reported LDH subgroup results.

### 3.2 Characteristics of included trials and quality evaluation

Of all trails, five trails were designed as phase III RCTs, two trails (CAPSTONE −1 and ASTRUM‐005) were first reported in 2022, and two trails (CASPIAN and KEYNOTE‐604) updated their results in 2022. The main characteristics of included trials were shown in Table 1. The median follow‐up ranged from 12.3 to 43.3 months. Only two trials (CASPIAN and ECOG‐ACRIN EA5161) were at high risk of performance bias and attrition bias. Quality evaluation of each trail was provided in Appendix S2.

**TABLE 1:** Summaries of included trails.

Trail (year)	Phase	Treatment	No. of patients	Median age (year)	Male (%)	Never smoke (%)	ECOG 0 (%)	BM (%)	LM (%)	PD‐L1 ≥ 1%	LDH > ULN	Median follow‐up (months)
IMpower133 (2021)^9^, ^11^	III	Atezo+EC	201	64	64.2	4.5	36.3	8.5	38.3	NR	NR	22.9
		EC	202		65.3	1.5	33.2	8.9	35.6			
CASPIAN (2022)^12^, ^13^, ^14^, ^15^	III	Durva+EP	268	63	70.9	8.2	36.9	10.4	40.3	NR	NR	39.4
		EP	269		68.4	5.6	33.5	10.0	38.7			
CAPSTONE‐1 (2022)^16^	III	Adebre+ EC	230	62	80.0	21.7	14.3	2.2	31.7	10.4	49.6	13.5
		EC	232		81.0	22.8	12.9	2.2	31.9	8.6	50.4	
KEYNOTE‐604 (2022)^17^, ^18^	III	Pem + EP	228	65	66.7	3.5	26.3	14.5	41.7	38.6	55.7	43.3
		EP	225		63.1	3.6	24.9	9.8	40.9	43.1	57.3	
ECOG‐ACRIN EA5161 (2020)^19^	II	Nivo+EP	80	NR	43.7	NR	28.7	NR	NR	NR	NR	NR
		EP	80		45.0	NR	30.0					
ASTRUM‐005 (2022)^20^	III	Serp+EC	389	61.1	81.5	20.8	18.3	12.9	25.4	15.9	NR	12.3
		EC	196		83.7	17.9	16.3	14.3	26.0	17.3		

Abbreviations: Adebre, Adebrelimab; Atezo, Atezolizumab; BM, brain metastases; Durva, Durvalumab; EC, etoposide plus carboplatin; ECOG, Eastern Cooperative Oncology Group; EP, etoposide plus carboplatin or cisplatin; LDH, lactate dehydrogenase; LM, liver metastases; Nivo, Nivolumab; NR, not reported; PD‐L1, programmed death ligand‐1; Pem, Pembrolizumab; Serp, Serplulima; ULN, upper limit of normal.

### 3.3 Outcomes of evaluation

**FIGURE 2:**
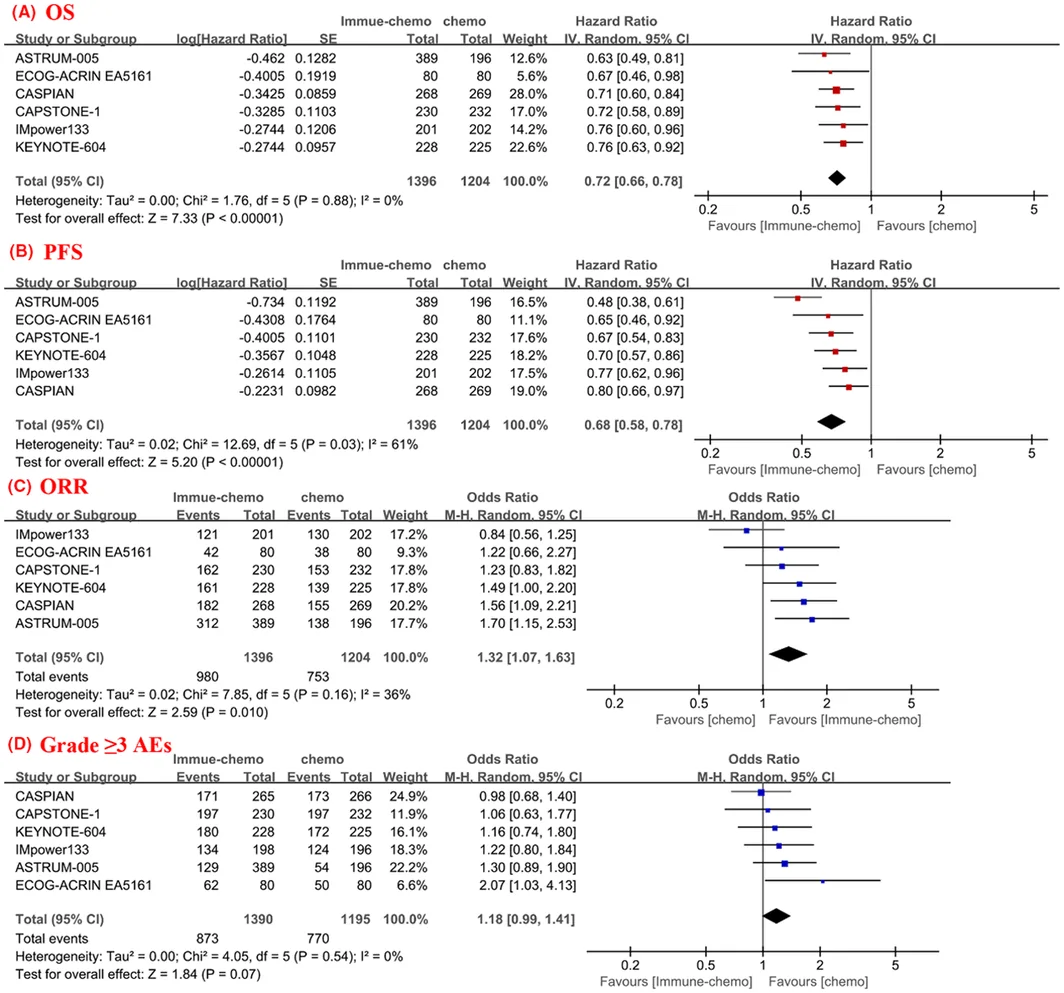
Forest plots for (A) overall survival (OS), (B) progression‐free survival (PFS), (C) overall response rate (ORR), and (D) Grade ≥3 adverse events (AEs).

**FIGURE 3:**
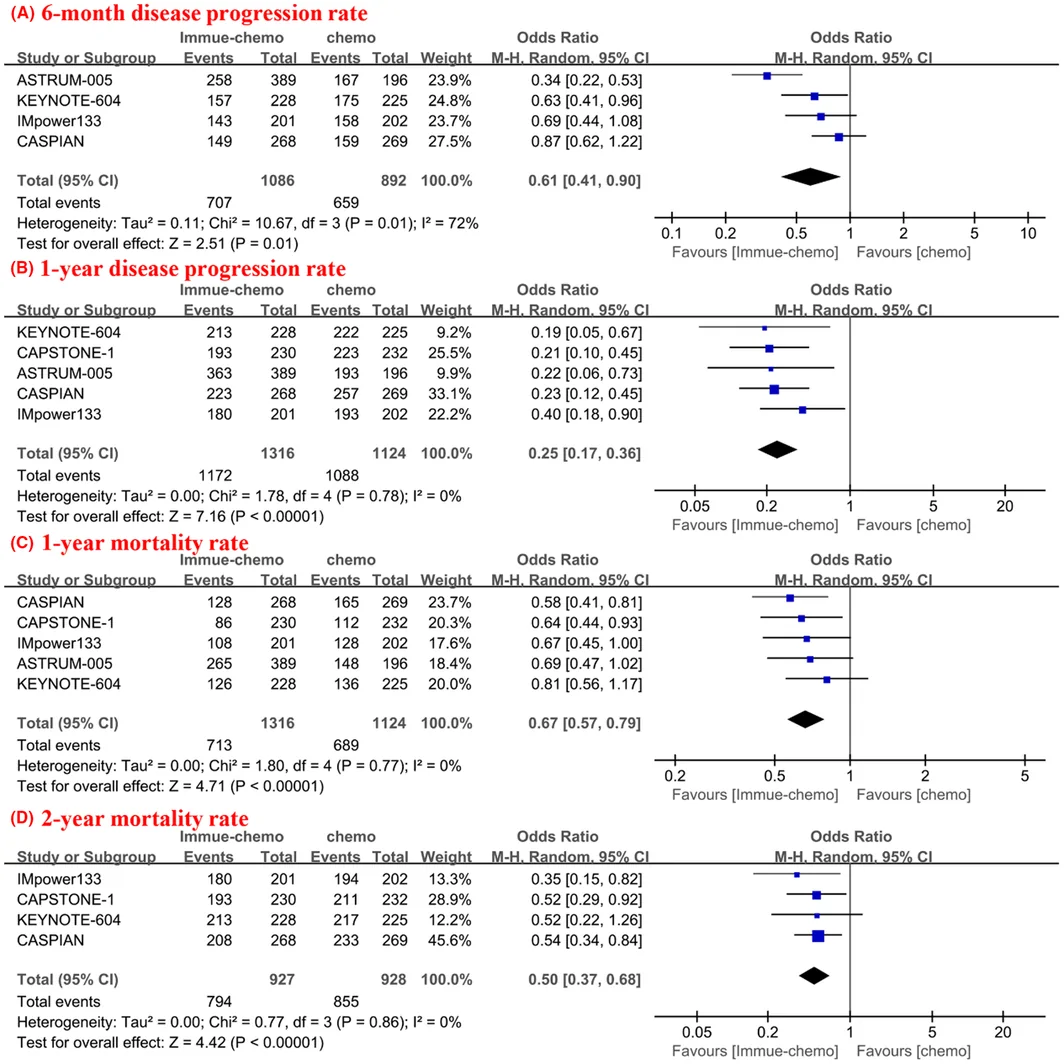
Forest plots for (A) 6‐month disease progression rate, (B) 1‐year disease progression rate, (C) 1‐year mortality rate, and (D) 2‐year mortality rate.

In comparison with chemotherapy, immunochemotherapy significantly improved ORR (OR 1.32, 95% CI 1.07–1.63; *p* = 0.01), PFS (HR 0.68, 95% CI 0.58–0.78; *p* < 0.001), and OS (HR 0.72, 96% CI 0.66–0.78; *p* < 0.001) without increasing Grade ≥3 AEs (OR 1.18, 95% CI 0.99–1.41; *p* = 0.07) (Figure 2). Compared with chemotherapy, immunochemotherapy reduced the 6‐month disease progression rate by 0.39 (95% CI 0.10–0.59; *p* = 0.01) and the 1‐year disease progression rate by 0.75 (95% CI 0.64–0.83; *p* < 0.001). First‐line immunochemotherapy significantly prolonged the survival of patients with ES‐SCLC and decreased the 1‐year mortality rate by 0.33 (95% CI 0.21–0.43; *p* < 0.001) and the 2‐year mortality rate by 0.50 (95% CI 0.32–0.63; *p* < 0.001) (Figure 3).

### 3.4 Subgroup analysis

**FIGURE 4:**
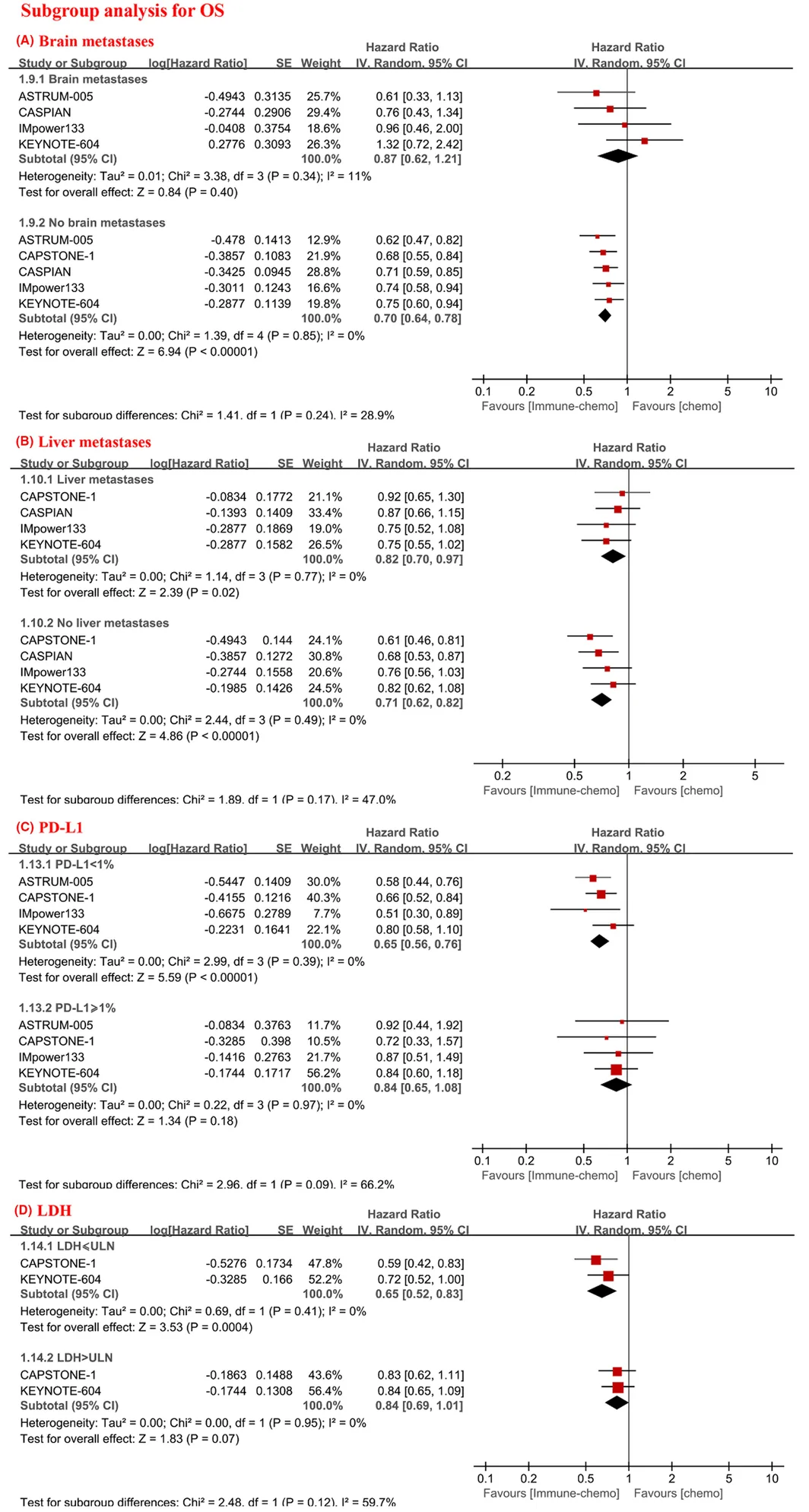
Forest plots for OS according to (A) brain metastases, (B) liver metastases, (C) PD‐L1, and (D) LDH. OS, overall survival; PD‐L1, programmed cell death ligand‐1; LDH, lactate dehydrogenase; immune‐chemo, immunochemotherapy; chemo, chemotherapy.

**FIGURE 5:**
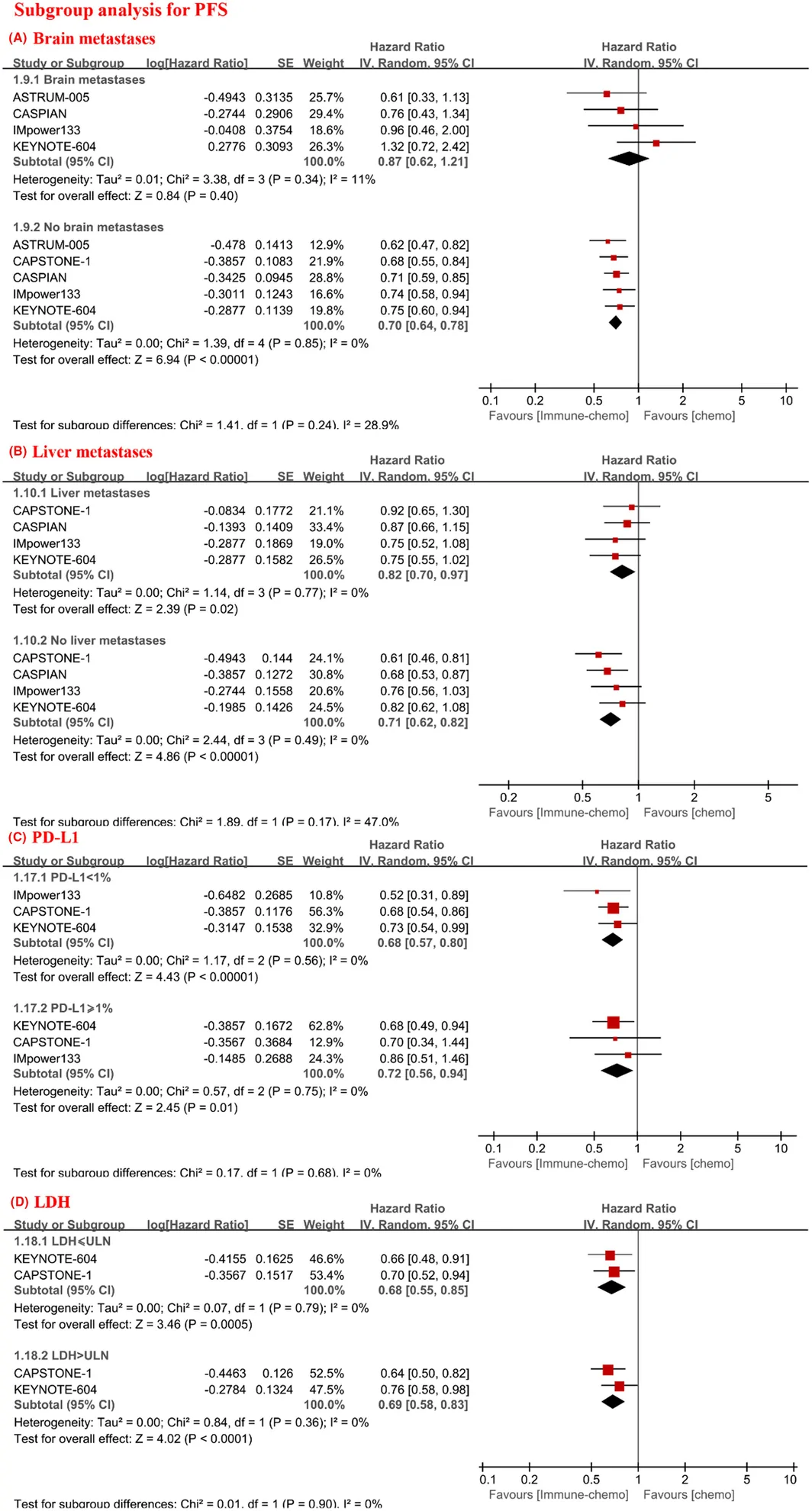
Forest plots for PFS according to (A) brain metastases, (B) liver metastases, (C) PD‐L1, and (D) LDH. PFS, progression‐free survival; PD‐L1, programmed cell death ligand‐1; LDH, lactate dehydrogenase; immune‐chemo, immunochemotherapy; chemo, chemotherapy.

Subgroup analysis was conducted stratified by brain metastases, liver metastases, PD‐L1, and LDH. Regarding OS, our findings indicated that immunochemotherapy was associated with improved survival compared with chemotherapy in the subgroups of liver metastases, no liver metastases, no brain metastases, PD‐L1 < 1%, and LDH < upper limit of normal (ULN) (all *p* < 0.05). However, patients did not get benefit from immunochemotherapy in the subgroups of brain metastases, PD‐L1 ≥ 1%, and LDH ≥ ULN (*p* > 0.05) (Figure 4). Regarding PFS, patients who received immunochemotherapy had more benefits than those who received chemotherapy in all subgroups except for the brain metastases subgroup with a *p*‐value of 0.34 (Figure 5).

### 3.5 Heterogeneity and publication bias

Overall, no significant heterogeneity was identified among included trials for OS (*I* ^2^ = 0%; *p* = 0.88), ORR (*I* ^2^ = 36%; *p* = 0.16), and Grade ≥3 AEs (*I* ^2^ = 0%; *p* = 0.54), while some heterogeneity existed in PFS (*I* ^2^ = 61%; *p* = 0.03) (Figure 2). For the 6‐month disease progression rate, some heterogeneity existed among trials (*I* ^2^ = 72%; *p* = 0.01), but no heterogeneity was observed in the disease progression rate at 1 year, and the mortality rate at 1 year and 2 years (all *I* ^2^ = 0% and *p* > 0.1) (Figure 3). Subgroup analysis showed no significant heterogeneity among trials (Figures 4 and 5). Publication bias was performed and the funnel plots showed symmetrical for OS and Grade ≥3 AEs, and little bias for PFS and ORR (Appendix S3).

### 3.6 Sensitivity analysis

The sensitivity analysis was performed to assess the robustness of the results. For OS, PFS, ORR, and Grade ≥3 AEs, the pooled results were not significantly affected although each trial was omitted separately, demonstrating the reliability of the results (Appendix S4).

## 4 DISCUSSION

Recently, immune checkpoint inhibitors, especially targeting PD‐(L)1, have led to significant regression of cancers with the mechanism of releasing the antitumor response of T cells.^21^ Immunotherapy‐based therapies as the first‐line treatment have significantly improved treatment efficacy and survival compared with chemotherapy alone in cancers.^22^, ^23^, ^24^ ES‐SCLC is considered refractory cancer with very rapidly progressive disease. The combination of PD‐(L)1 and chemotherapy yields one therapeutic breakthrough in recent years.

This meta‐analysis systematically reviewed published data comparing PD‐(L)1 inhibitor plus chemotherapy versus chemotherapy as first‐line treatment for patients with ES‐SCLC. The pooled results showed that immunochemotherapy not only improved the short‐term outcomes of ORR and PFS but also improved the long‐term survival rate without causing more severe adverse effects. In this study, we first evaluated the PFS rate (at 6 months and 1 year) and the OS rate (at 1 year and 2 years), our results indicated the greater benefit of PD‐(L)1 inhibitor plus chemotherapy than chemotherapy. Next, subgroup analysis showed that patients who received immunochemotherapy had PFS benefits regardless of liver metastases, PD‐L1, and LDH, however, patients with brain metastases, PD‐L1 ≥ 1%, and LDH ≥ ULN did not get OS benefits.

The previous study^25^ compared immunochemotherapy and chemotherapy in first‐line treatment of ES‐SCLC, but due to the lack of data and short‐term follow‐up, only four RCTs were included, the outcomes of PFS rate (6 months, 1 year), OS rate (1 year, 2 years), and subgroup analysis for PD‐L1 and LDH were not performed. Therefore, we systemically conducted this meta‐analysis to determine whether immunochemotherapy was superior to chemotherapy in the first‐line treatment of ES‐SCLC, especially for brain metastases, liver metastases, PD‐L1, and LDH subgroups.

The study has several limitations. Firstly, due to a lack of data, only six RCTs were included, of which five were phase III studies and one was a phase II study. Secondly, the duration of follow‐up is known to affect survival outcomes, and in this study, five trials had a median follow‐up of 12.3–43.3 months, while one trial did not report follow‐up. Thirdly, the study designs were not consistent, with one trial (CASPIAN) having three arms (PD‐L1 inhibitor + chemotherapy + tremelimumab vs. PD‐L1 inhibitor + chemotherapy vs. chemotherapy), while the other five trials had two arms (PD‐1/PD‐L1 inhibitor + chemotherapy vs. chemotherapy). However, we only extracted the data comparing PD‐(L)1 inhibitor plus chemotherapy with chemotherapy. Finally, different characteristics of included trials and different regiments may affect the final results, this study included three trials using PD‐1 inhibitors (pembrolizumab, nivolumab, and serplulimab) and three trials using PD‐L1 inhibitors (durvalumab, atezolizumab, and adebrelimab). Despite the above limitations, this study still demonstrated that the combination of PD‐(L)1 inhibitor and chemotherapy as first‐line treatment could improve the efficacy and prognosis of patients with ES‐SCLC.

## 5 CONCLUSION

The combination of PD‐(L)1 inhibitor and chemotherapy as first‐line treatment has been found to improve the efficacy and prognosis of patients with ES‐SCLC without causing more serious side effects. However, further research is required to validate these findings.

## AUTHOR CONTRIBUTIONS

**Jiangyue Lu:** Writing – original draft (lead); writing – review and editing (lead). **Xiao Lei:** Writing – review and editing (supporting). **Pei Zhang:** Writing – review and editing (equal). **Lehui Du:** Writing – review and editing (equal). **Zhibo Zhang:** Data curation (lead); formal analysis (lead); writing – review and editing (lead). **Baolin Qu:** Writing – review and editing (equal).

## CONFLICT OF INTEREST STATEMENT

All authors declare no conflict of interest.

## Supporting information


Appendix S1.

## Data Availability

Data sharing is not applicable to this article as no new data were created or analyzed in this study.
