# Supplementary material for: Meta‐analysis of PD‐(L)1 inhibitor plus chemotherapy versus chemotherapy as first‐line treatment in extensive‐stage small‐cell lung cancer
Source: Cancer Med. 2023 Aug 11;12(17):17924–33. doi: 10.1002/cam4.6433 (PMC10524002; doi:10.1002/cam4.6433)
Supplement: Supplementary file 1 — Appendix S1. [file CAM4-12-17924-s001.docx]

**Appendix**

1. **Search strategy.**
2. **Quality evaluation of included trails.**
3. **Funnel plots of included trails.**
4. **Plots of sensitive analysis by excluding one trial each time and the pooled estimates for the test of the study.**

**Appendix 1. Search Strategy**

**PubMed**

| **No.** | **Query** | **Results** |
| --- | --- | --- |
| #1 | “immune checkpoint” OR "programmed cell death-1" OR "programmed cell death ligand-1" OR PD-1 OR PD-L1 OR nivolumab OR opdivo OR pembrolizumab OR keytruda OR atezolizumab OR tecentriq OR durvalumab OR imfinzi OR avelumab OR adebrelimab OR serplulimab OR cemiplimab OR envafolimab OR pidilizumab | 48799 |
| #2 | chemotherapy OR carboplatin OR cisplatin OR cis-platinum OR platinum OR etoposide | 3888390 |
| #3 | carcinoma, small cell lung [MeSH] OR sclc | 11945 |
| #4 | “extensive-stage” OR “extensive stage” OR “extensive-disease” OR “extensive disease” OR “extensive” | 380815 |
| #5 | "first-line" OR "untreated" OR "treatment naive" OR "chemo naive" OR "front line" | 313423 |
| #6 | randomized controlled trial [Title/Abstract] OR RCT[Title/Abstract] OR controlled clinical trial [Title/Abstract] OR randomized [Title/Abstract] OR randomly [Title/Abstract] OR trial [Title/Abstract] | 1373213 |
| #7 | #1 AND #2 AND #3 AND #4 AND #5 AND #6 | 63 |

**Embase**

| **No.** | **Query** | **Results** |
| --- | --- | --- |
| #1 | ‘immune checkpoint’ OR 'programmed cell death-1’ OR 'programmed cell death ligand-1' OR PD-1 OR PD-L1 OR nivolumab OR opdivo OR pembrolizumab OR keytruda OR atezolizumab OR tecentriq OR durvalumab OR imfinzi OR avelumab OR adebrelimab OR serplulimab OR cemiplimab OR envafolimab OR pidilizumab | 109587 |
| #2 | 'chemotherapy':ab,ti OR 'carboplatin':ab,ti OR 'cisplatin':ab,ti OR 'cis-platinum':ab,ti OR 'platinum':ab,ti OR 'etoposide':ab,ti | 778235 |
| #3 | 'small cell lung cancer'/exp OR 'sclc':ab,ti | 36383 |
| #4 | 'extensive-stage':ab,ti OR 'extensive stage':ab,ti OR 'extensive-disease':ab,ti OR 'extensive disease':ab,ti | 6914 |
| #5 | 'first-line':ab,ti OR 'treatment naive':ab,ti OR 'chemo naive':ab,ti OR 'front line':ab,ti | 218654 |
| #6 | 'randomized controlled trial':ab,ti OR 'RCT':ab,ti OR 'controlled clinical trial':ab,ti OR 'randomized':ab,ti OR 'randomly':ab,ti OR 'trial':ab,ti | 1954906 |
| #7 | #1 AND #2 AND #3 AND #4 AND #5 AND #6 | 190 |

**Cochrane Library**

| **No.** | **Query** | **Results** |
| --- | --- | --- |
| #1 | immune checkpoint OR programmed cell death-1 OR programmed cell death ligand-1 OR PD-1 OR PD-L1 OR nivolumab OR opdivo OR pembrolizumab OR keytruda OR atezolizumab OR tecentriq OR durvalumab OR Imfinzi OR avelumab OR adebrelimab OR serplulimab OR cemiplimab OR envafolimab OR pidilizumab | 9413 |
| #2 | chemotherapy OR carboplatin OR cisplatin OR cis-platinum OR platinum OR etoposide | 100505 |
| #3 | “small cell lung cancer” OR sclc | 16612 |
| #4 | “extensive-stage” OR “extensive stage” OR “extensive-disease” OR “extensive disease” OR “extensive” | 13949 |
| #5 | first-line OR untreated OR treatment naive OR chemo naive OR front line | 60764 |
| #6 | "randomized controlled trial" OR RCT OR "controlled clinical trial" OR randomized OR randomly OR trial | 1532441 |
| #7 | #1 AND #2 AND #3 AND #4 AND #5 AND #6 | 149 |

**
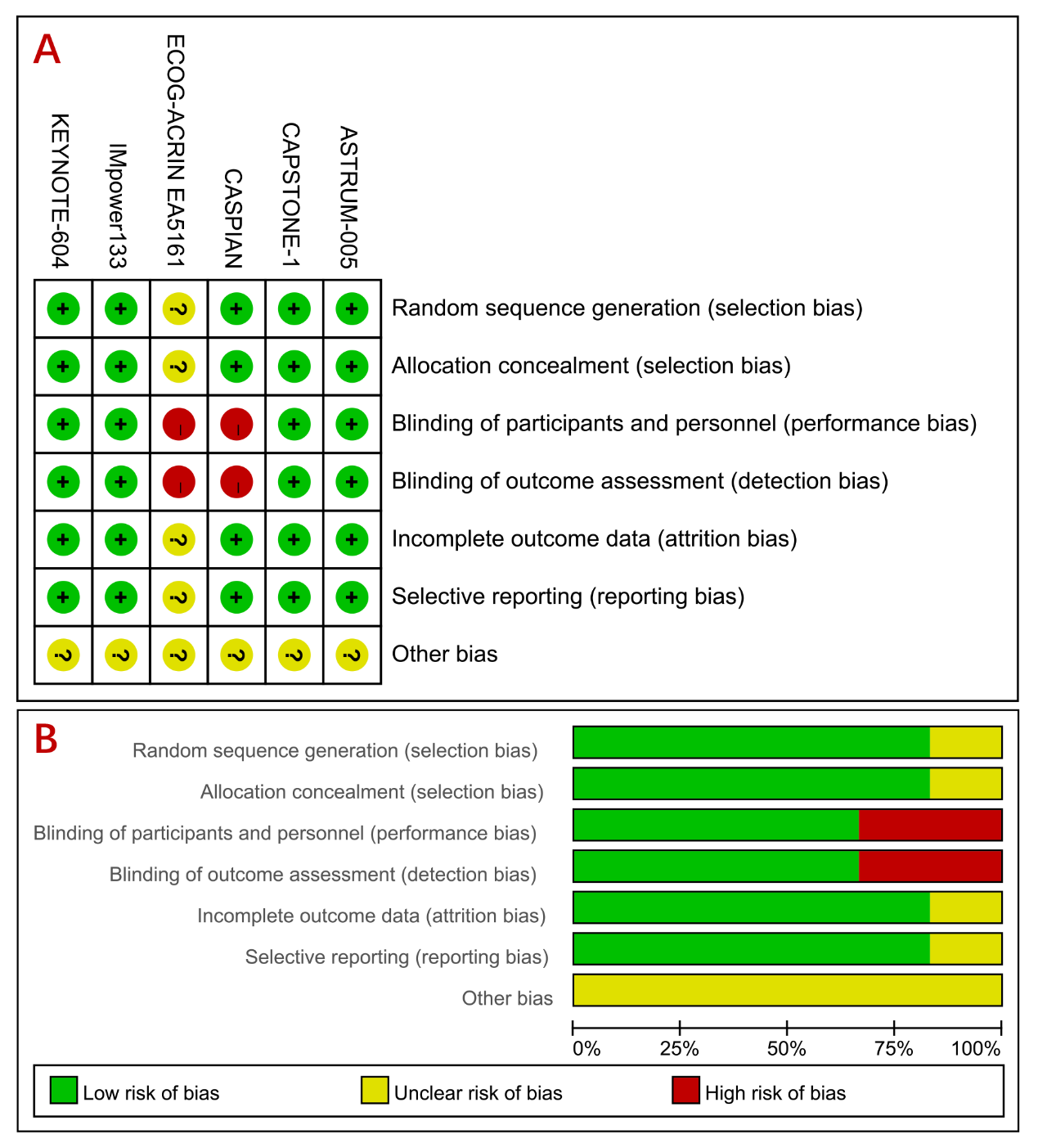
**

**Appendix 2. Quality evaluation of included trails.** (A) Risk of bias for each study. (B) Bar chart comparing percentage risk of bias for each study.

**
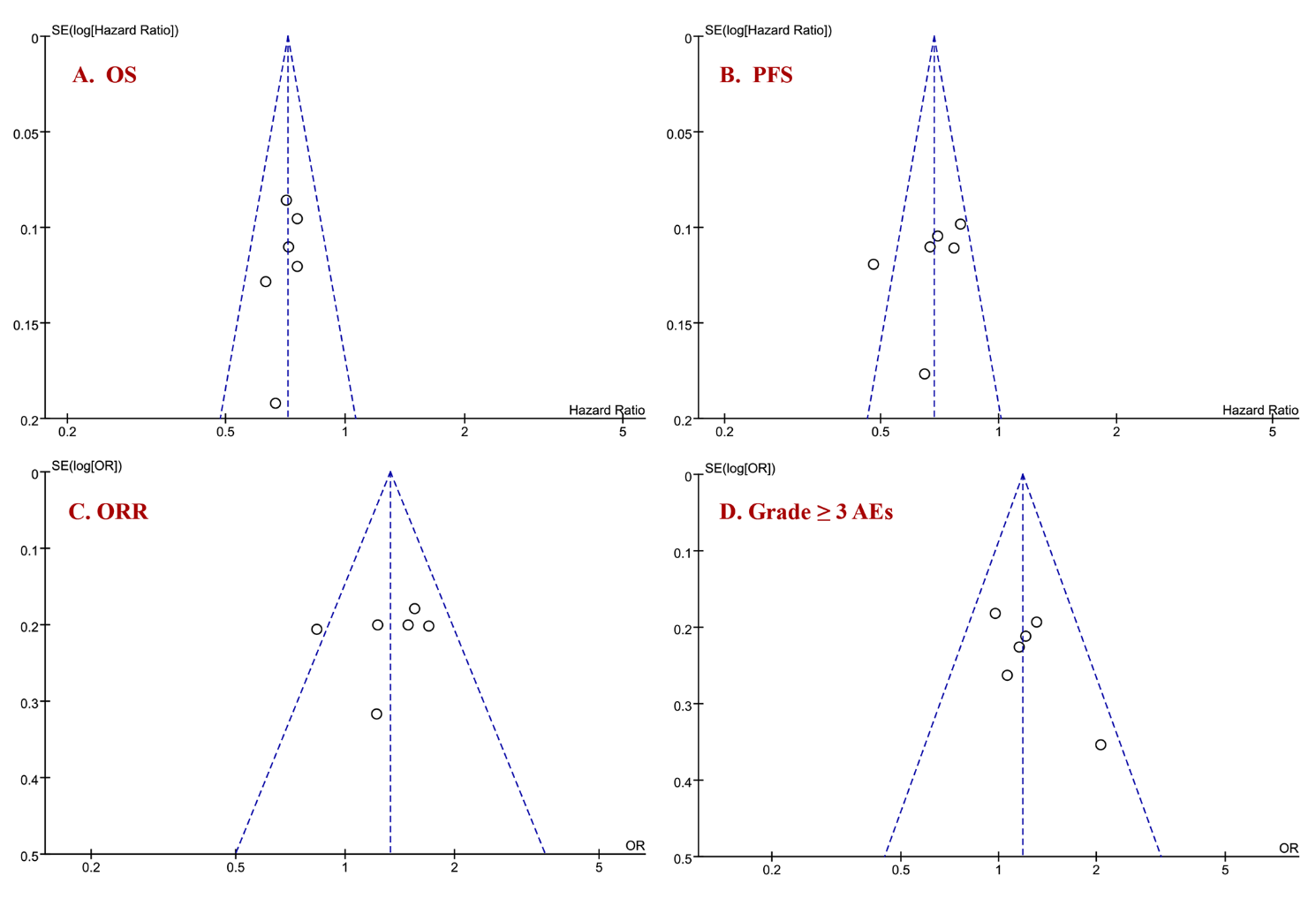
**

**Appendix 3. Funnel plots** **of included trails.** (A) for overall survival (OS); (B) for progression-free survival (PFS); (C) for overall response rate (ORR); (D) for grade ≥3 adverse events (AEs).

**
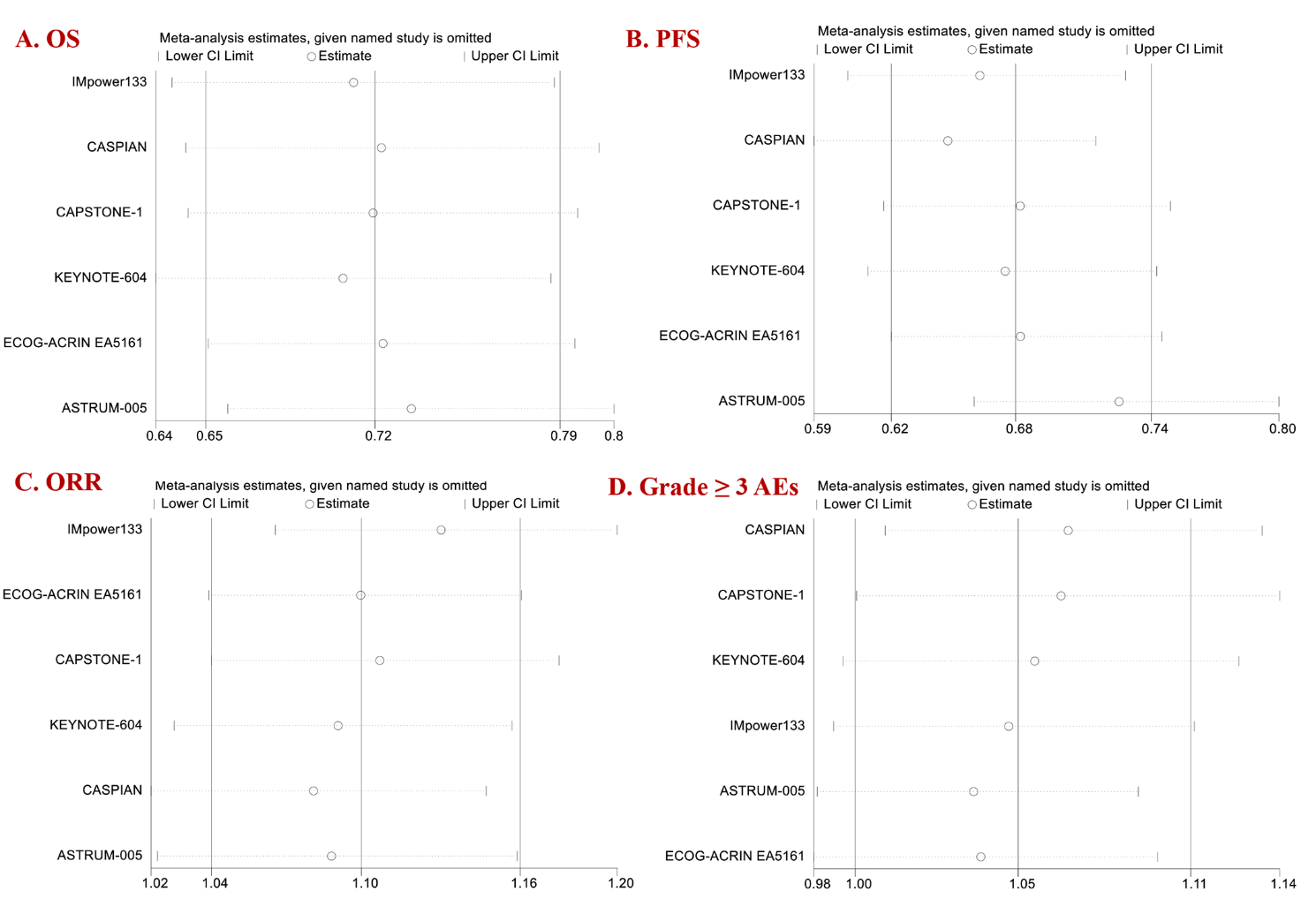
**

**Appendix 4. Plots of sensitive analysis by excluding one trial each time and the pooled estimates for the test of the study.** (A) overall survival (OS); (B) progression-free survival (PFS); (C) overall response rate (ORR); (D) grade ≥ 3 adverse events (AEs).
